# Development of Oleic Acid Composite Vesicles as a Topical Delivery System: An Evaluation of Stability, Skin Permeability, and Antioxidant and Antibacterial Activities

**DOI:** 10.3390/molecules31010122

**Published:** 2025-12-29

**Authors:** Xinyue Ma, Qinqing Zhang, Ying Yang, Yuqi Zhan, Xiangyu Zhang, Yanli Zhao, Jinlian Li, Dongmei Wu

**Affiliations:** 1 College of Pharmacy, Jiamusi University, Jiamusi 154007, China; 18249143626@163.com (X.M.); zhangqinqing2000@163.com (Q.Z.); 18249574539@163.com (Y.Y.); 14743822001@163.com (Y.Z.); zhaoyanli@jmsu.edu.cn (Y.Z.); lijinlian@jmsu.edu.cn (J.L.); 2 Heilongjiang Provincial Key Laboratory of New Drug Development and Pharmacotoxicological Evaluation, Jiamusi University, Jiamusi 154007, China

**Keywords:** fatty acid vesicle, topical delivery system, stability, skin permeability, antioxidant activity, antibacterial activity

## Abstract

Fatty acid vesicles (FAVs) are promising nanocarriers, but their application is limited by a narrow, alkaline pH formation window that mismatches the weak acidity of physiological environments, such as skin. To overcome this, we developed composite vesicles using oleic acid (OA) and the non-ionic surfactant Tween 40 (TW40). pH titration confirmed that the OA/TW40 system successfully broadened the vesicle formation window from 8.2–10.08 to 3.1–7.2, aligning it with the physiological pH range. The bioactive flavonoid luteolin (LUT) was efficiently encapsulated into these OA/TW40-FAVs, achieving a high encapsulation efficiency (EE) of 87.13% and a drug loading capacity (DLC) of 9.58. The formulation demonstrated superior topical delivery performance: the cumulative transdermal flux (933.08 µg·cm^−2^) and skin retention (68.18 µg·cm^−2^) were both approximately double that of the free LUT solution. Furthermore, the OA/TW40/LUT-FAVs provided sustained drug release and exhibited synergistically enhanced antioxidant and antimicrobial activities compared to free LUT or blank vesicles. Collectively, these findings establish OA/TW40 composite vesicles as a robust and efficient nanoplatform for the topical delivery of bioactive compounds.

## 1. Introduction

The inherent limitations of bioactive ingredients, such as poor aqueous solubility, chemical instability, and low penetration, can be addressed using functional delivery systems. Self-assembly has emerged as a prominent technology for preparing organized nanostructures, including micelles, vesicles, and hydrogels, as it enables spontaneous formation without external stimuli. The pursuit of economical production processes for these nanostructures has positioned self-assembly technology as a key area of development [1]. As an alternative to traditional delivery systems like liposomes and synthetic polymers, fatty acid vesicles (FAVs) have recently gained attention. Compared to liposomes, FAVs offer distinct advantages for dermal delivery: they are composed of fatty acids (e.g., oleic acid) and non-ionic stabilizers—as opposed to the phospholipids and cholesterol in liposomes—and maintain stability primarily through fatty acid intermolecular hydrogen bonding and hydrophobic interactions, which may offer formulation benefits. FAVs also exhibit higher pH responsiveness, suggesting potential for adaptation to different physiological or formulation environments, along with anticipated good skin biocompatibility and strong affinity for lipid-soluble actives, enabling efficient encapsulation. In contrast, liposomes are predominantly suited for hydrophilic actives and can be unstable in oil-rich matrices [2]. Their selective permeability and dynamic properties have made FAVs a subject of growing scientific interest. In living systems, fatty acids serve not only as an energy source but also as essential structural components of cell membranes, influencing diverse cellular processes [3,4,5]. FAVs, which self-assemble into structures featuring an aqueous core enclosed by a hydrophobic bilayer, represent ideal nanocontainers for encapsulating and controlling the release of compounds of varying polarity [6]. They are thus considered to have broad application potential in pharmaceuticals, topical delivery, gene delivery, and as nanoreactors [7,8,9,10].

However, the two inherent limitations of FAVs limit their research and application. To begin with, the pKa of the fatty acids of which the vesicle consists comes mostly from a weakly acidic to neutral pH range, which is observed in most biological systems [11]. Another reason for the above is that the solution pH range, effective for vesicle stability, is only about 1 pH unit, hence, the vesicles become destabilized with the change in pH. The limitations are shown in their pH dependence and sensitivity. The addition of surfactants is a promising strategy for simultaneous migration regulation and pH range widening. According to Walde and Trishool [12], incorporation of an equimolar amount of sodium dodecyl benzene sulfonate (SDBS) shifts the pH window for vesicle formation of decanoic acid vesicles to a more acidic range, extending it to between pH 4.3 and 7.51. Caschera et al. [13] suggested that the pH range for vesicle formation was found to be 5.4–8.6 after the addition of DTAB. These collective findings indicate that the addition of surfactants to fatty acids can expand and shift the pKa window for vesicle formation. The important long-chain fatty acid, oleic acid (OA), self assembles to give oleic acid vesicles. Vesicles of this nature have been proven to be desirable and effective carriers for active ingredients in the cosmetic sector [14]. The stability of the vesicle formed by fatty acid may be improved by the addition of surfactant Tween 40 (TW40). Moreover, it can adjust to the pH microenvironment that human skin surface needs [15]. In the end, this provides great help to the extension of application scenarios of this carrier system.

Luteolin (LUT), a natural flavonoid broadly occurring in plants, exhibits numerous bioactivities, such as antioxidant, anti-inflammatory, and antibacterial, as well as antitumor activities [16,17,18]. It has been widely utilized in cosmetics and pharmaceuticals, as well as in food production, for example, as a natural food preservative instead of synthetic counterparts for enhancing food shelf life [19]. Nevertheless, its application remains restricted owing to poor solubility in water, insufficient chemical stability, low oral availability, as well as a high rate of metabolism [20]. Zhao, Y et al. [21] employed fatty acid vesicles as a delivery system for the poorly soluble drug Tre, which resulted in a high EE of 84.26%. Tre-FAV had a slow release behavior as well as high skin penetration and retention capability. Cristiano et al. [22] performed physicochemical characterization, as well as in vitro/in vivo studies, on unsaturated fatty acid vesicles loading ammonium glycyrrhizate, which resulted in a high EE of 80%. LUT-loaded composite fatty acid vesicles’ self-assembly may serve as a method for effective encapsulation as well as dermatologically localized delivery of LUT.

This study employed OA as a model fatty acid and TW40 as a structural component to prepare blank composite vesicles (OA/TW40-FAV) via self-assembly. The primary objective was to reduce the vesicle’s pH dependency and tailor its formation window to better align with physiological conditions. Luteolin (LUT) was used as a model bioactive compound, and the resulting composite vesicles were systematically evaluated for encapsulation efficiency (EE), drug loading capacity (DLC), release behavior under various conditions, skin penetration, antioxidant activity, and antimicrobial efficacy. These investigations were conducted to assess their potential as a topical delivery vehicle. The findings demonstrated that the composite vesicles efficiently encapsulated LUT, enhanced its bioavailability, and established the stable OA/TW40-FAV as a promising carrier system for dermal applications.

## 2. Results and Discussion

### 2.1. Establishment of the OA/TW40-FAV pH Window

**Figure 1:**
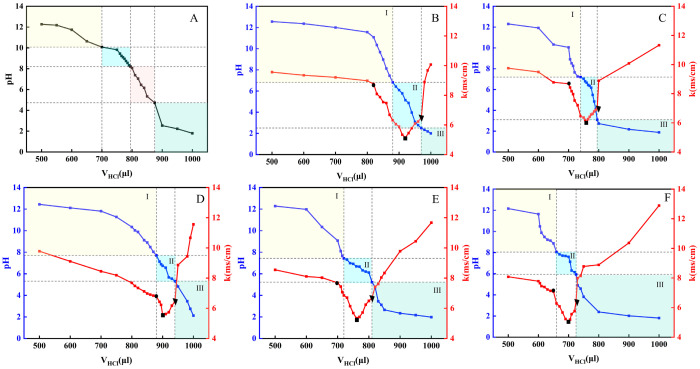
(**A**) pH titration curve of oleic acid vesicles at room temperature. (**B**–**F**) The pH titration curves of the OA/TW40 composite solution system (R = 5:1; 10:1; 15:1; 20:1; 25:1). The circular markers represent the micelle-to-vesicle transition, the rectangular markers indicate the stable vesicle region at the conductivity minimum, and the triangular markers denote the vesicle-to-emulsion transition.

Due to the solubilizing effect of surfactants on the vesicle system, the original pH titration method cannot be employed to determine the pH window range. In this study, a combination of pH titration and conductivity measurement was adopted to identify the pH window of OA composite vesicles [23]. In Figure 1A, the initial pH range for vesicle formation by OA alone spanned from 8.2 to 10.08, covering an interval of only 1.88 units. The pH of the human skin surface typically ranges between 4.5 and 6.5. Therefore, the pH window for vesicle formation by OA does not align with this physiological range. Consequently, TW40 was incorporated with OA, and varying molar ratios of OA/TW40-FAV were prepared by adjusting the amount of TW40 added. With the gradual dropwise addition of 0.1 mmol/L HCl (Figure 1B–F), the pH decreased continuously, accompanied by a rapid decline in conductivity. After neutralization of excess NaOH by HCl, deprotonated carboxylate ions (-COO^−^) of OA formed hydrogen bonds with hydroxyl groups (-OH) of TW40, which resulted in the lowest conductivity, indicating OA/TW40-FAV formation. When HCl was further added beyond this point, pH was further lowered, but conductivity increased substantially. During this whole titration, there were four phase transformations in the system: micelles, vesicles, emulsions, and oil–water separation. Correspondingly, the appearance of the solution changed from transparent colorless to blue milky light, then milky turbid, and eventually formed phase separation. For different molar ratios, in Table 1, the pH window changed and widened. Interestingly, pH windows seen at molar ratios of 10:1 and 5:1 matched well with the living system physiological pH range. Considering the TW40 added amount, 10:1 was chosen for further experiments, which resulted in a pH window of 3.1–7.2 for OA/TW40-FAV. This window aligns well with the physiological pH of skin, thereby enhancing the formulation stability and compatibility of the system, which supports its potential for dermal applications.

### 2.2. Mechanism of OA/TW40-FAV Formation and Migration Broadening pH Window

**Figure 2:**
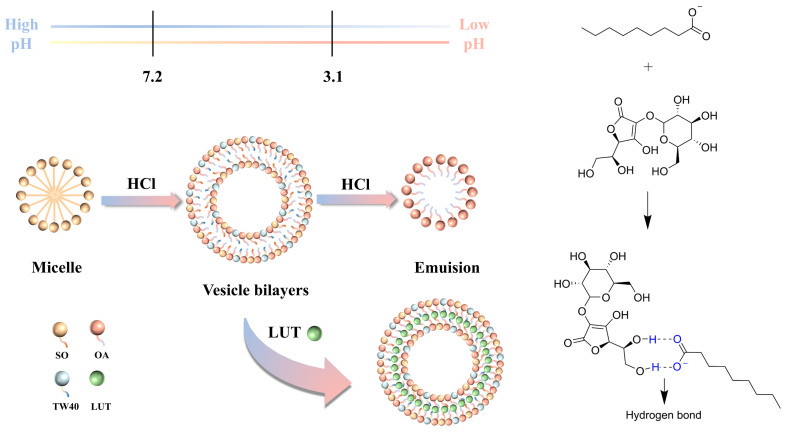
Structural transformation schematic diagram of OA/TW40/LUT composite vesicle system.

Figure 2 illustrates the formation mechanism of OA/TW40 composite vesicles and the corresponding broadening of the pH window, with direct morphological validation provided by the TEM images. Under alkaline conditions, deprotonated OA carboxylate ions stabilize micelle formation through electrostatic repulsion. This represents the narrow native pH window of pure OA systems. As the system is gradually acidified, hydrophobic interactions drive the aggregation of OA alkyl chains with the hydrophobic core of TW40, while hydrogen bonding between OA carboxyl groups and TW40 hydroxyl groups (confirmed by the characteristic C=O peak shift in FT-IR analysis) synergistically guides and stabilizes the assembly of the vesicle bilayer. Critically, this molecular synergy enables the maintenance of stable vesicular structures across an expanded pH range from 3.1 to 7.2, effectively broadening the practical application window beyond the limited range of pure OA vesicles [24]. The corresponding TEM images at this stage clearly show well-defined spherical vesicle structures. Finally, when the environmental pH drops below a critical value, excessive protonation disrupts both the hydrogen bonding network and molecular ordering, leading to vesicle disintegration and transition to an emulsion state. This extended stability window, coupled with the visual evidence of phase transitions, provides intuitive and reliable experimental evidence for understanding the molecular self-assembly mechanism and enhanced system stability under pH regulation. The demonstrated pH adaptability significantly improves the formulation’s potential for physiological applications where pH variations occur.

### 2.3. Characterization of OA/TW40-FAV

#### 2.3.1. Particle Size Analysis

**Figure 3:**
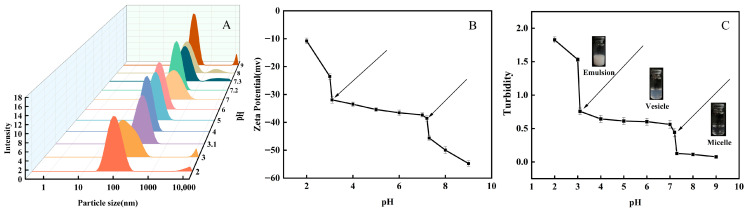
(**A**) Particle size, (**B**) zeta potential, (**C**) appearance and turbidity of OA/TW40 composite solution system with different pHs (R = 10:1). Data are expressed as the mean ± standard deviation.

This work utilizes dynamic light scattering (DLS) to analyze self-assembly characteristics of OA/TW40 composite vesicles over a range of pH values [25]. In Figure 3A, at a pH value higher than 7.2, a bimodal distribution occurs, with a secondary peak intensity increasing simultaneously with pH. Under high pH conditions, particle surfaces possess a negative charge, which increases electrostatic repulsion, promotes aggregation, and produces composite micelles. Within the pH window of 3.1–7.2, the system exhibited good dispersion quality with polydispersity index (PDI) values ranging between 0.273 ± 0.004 and 0.291 ± 0.005, indicating a monomodal and homogeneous size distribution. In this pH range, a single peak distribution characteristic of a state of good dispersion results (Table S1), with mean OA/TW40-FAV vesicle sizes between 100 and 200 nm. Under such conditions, hydrogen bonds between deprotonated carboxylate ions of the OA/TW40-FAV system and protonated carboxylic acid groups occur in a 1:1 mixture. When pH goes below 3.1, a distinct bimodal distribution reappears, with a sharp secondary peak. Correspondingly, the PDI values exceed 0.3, reflecting the increased heterogeneity and coexistence of multiple aggregates under these conditions. With a low pH, a low level of dissociation of carboxylate groups occurs, with a low negative surface charge, which destroys strong electrostatic repulsion, promotes phase separation of water from oil, and causes rupture of vesicles in the system.

#### 2.3.2. Zeta Potential Analysis

Zeta potential is a key parameter for measuring the stabilities of dispersion systems, such as colloids and suspensions. Zeta potential magnitude directly indicates repulsive electrostatic force strength between particles, hence its influence on the aggregation or dispersion propensities of a system. In Figure 3B and Table S2, zeta potentials at various pH levels were always negative, with absolute zeta potential value increasing with increasing pH. Above a pH of 7.2, extensive ionization produces -COO^−^, which confers a negative charge on particle surfaces. This increases electrostatic repulsion, enhancing composite micelle formation, as represented by high absolute zeta potential values. For pH between 3.1 to 7.2, vesicle zeta potential is constant between −30 mV and −40 mV, explained by a stable charge distribution enforced by hydrogen bonding [26]. For pH below 3.1, protonation suppresses particle surface charge, compacting the bilayer to its utmost extent and decreasing electrostatic repulsion to approximately zero. This situation causes vesicle disruption, as indicated by a low absolute value of zeta potential.

#### 2.3.3. Turbidity Analysis

In this investigation, the turbidity technique was utilized to examine the aggregation phase behavior of the OA/TW40 composite solution system over various pH ranges. With a pH value beyond 7.2 (Figure 3C and Figure S1 and Table S3), extensive deprotonation elevates hydrophilicity, facilitating the development of minute micelles with feeble light scattering. Consequently, low turbidity results, making the solution colorless and transparent [27]. With a pH range of 3.1–7.2, partial ionization establishes a hydrophilicity–hydrophobicity balance that favors vesicle development. Such vesicles scatter light moderately, giving intermediate turbidity with a blue milky appearance of light for the solution. With a decrease in pH below 3.1, molecular protonation increases hydrophobicity, which results in the overlapping of molecules with ensuing large emulsion development. This results in destruction of the bilayer, strong light scattering, high turbidity, with a milky white appearance or phase separation between water and oil in the solution.

#### 2.3.4. Transmission Electron Microscopy (TEM) Observations

**Figure 4:**
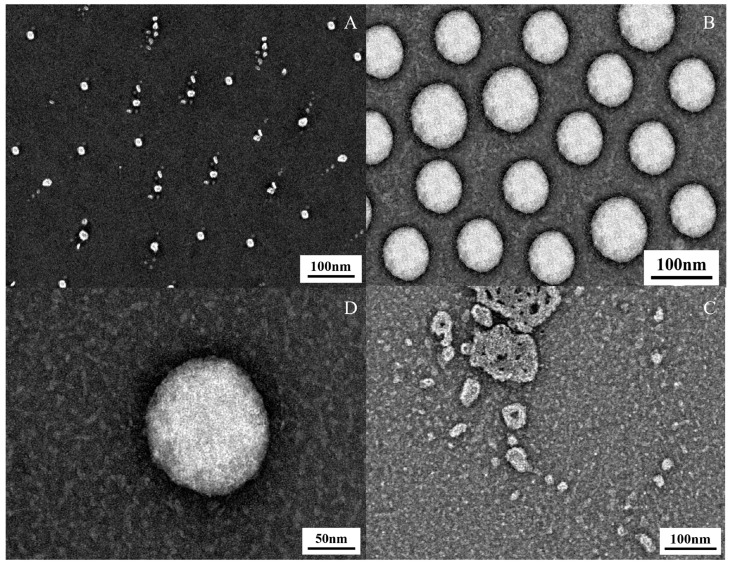
(**A**–**D**) TEM of OA/TW40 composite solution system. (**A**) pH = 9, (**B**) pH = 5.5, (**C**) pH = 3, and (**D**) microtransmission map at 50 nm at pH 5.5.

Figure 4 presents the TEM micrographs of the OA/TW40 composite solution under different pH conditions. As observed from the TEM images, vesicular structures are formed in the sample with a pH of 5.5, whereas no vesicles are detected in the samples at pH 3 and pH 9. It has been reported that pure oleic acid vesicles exhibit a pH stability window of 8.2–10.08, while the OA/TW40 composite vesicles, as demonstrated in our previous work, significantly expand this pH range to 3.1–7.2—a range that aligns with the physiological pH environment. Thus, the TEM results further validate the accuracy of the pH stability window for the OA/TW40 composite vesicles.

#### 2.3.5. FT-IR Analysis of OA/TW40 Composite Solution System

**Figure 5:**
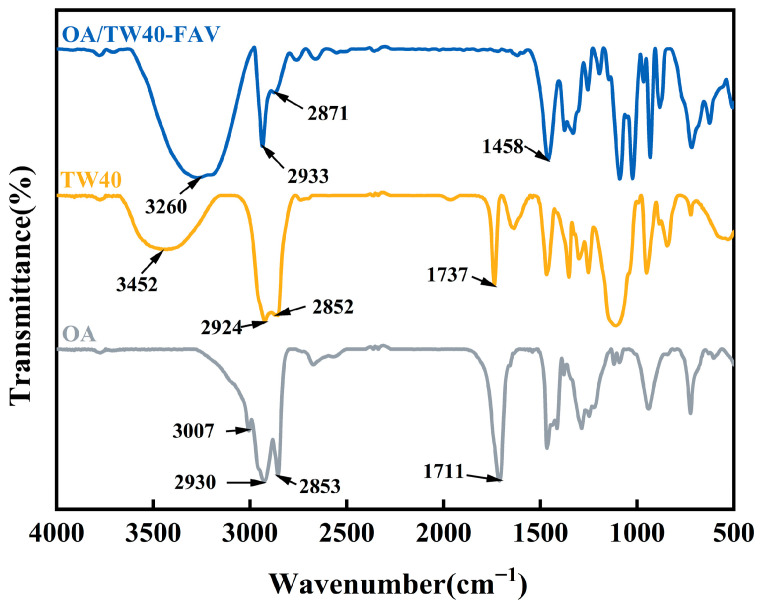
The FT-IR spectra of OA, TW40, OA/TW40-FAV.

Figure 5 presents the FT-IR spectra of OA, TW40, and the OA/TW40-FAV composite solution. The peak at 1711 cm^−1^ in the OA spectrum is assigned to the C=O stretching vibration of the carboxyl group, while the broad band at 3452 cm^−1^ in the TW40 spectrum corresponds to the O–H stretching vibration. In the OA/TW40-FAV composite solution, the characteristic carboxylic acid C=O stretching vibration of OA at 1711 cm^−1^ disappears. A new prominent band emerges at 1458 cm^−1^. Based on its position and the recent literature [28], this band is definitively assigned to the symmetric stretching vibration (*v*_s_) of the deprotonated carboxylate group (-COO^−^) of OA, as this vibrational mode typically occurs in the range of 1400–1460 cm^−1^, while the asymmetric stretching vibration (*v*_as_) is generally observed at 1550–1610 cm^−1^. This indicates the formation of OA carboxylate ions in the system. Simultaneously, the broad O–H stretching band of TW40 shifts to 3260 cm^−1^, confirming the establishment of hydrogen bonds between the deprotonated carboxylate groups (-COO^−^) of OA and the hydroxyl groups (-OH) of TW40 [29]. The characteristic peaks at 2924 cm^−1^ and 2864 cm^−1^ are attributed to the asymmetric and symmetric stretching vibrations of CH_2_ groups, respectively. The decreased intensity of these vibrations in the OA/TW40-FAV composite, compared to the individual components, reflects tighter packing of the alkyl chains and a more ordered hydrophobic core. These spectral changes collectively confirm that the formation of OA/TW40-FAV is driven by hydrogen bonding between the hydrophilic head groups and facilitated by the enhanced ordering of the hydrophobic chains.

### 2.4. Stability Analysis of OA/TW40-FAV

#### 2.4.1. Temperature Stability

**Figure 6:**
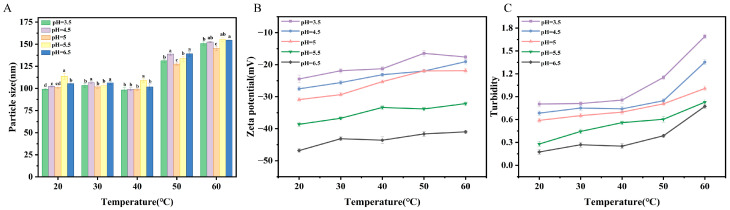
(**A**) Variations in particle size, (**B**) zeta potential and (**C**) turbidity of OA/TW40-FAV across a range of temperature (20–60 °C) at pH = 3.5, 4.5, 5, 5.5, 6.5. Data are expressed as the mean ± standard deviation. Different superscripts indicate statistical differences (*p* < 0.05).

Temperature stability has emerged as a key research area owing to its profound influence on structural dynamics and functional integrity, as well as possible applicability. Particle size remained substantially unaltered and stable while temperatures were increasing. Figure 6A–C exhibit a decreasing trend for absolute zeta potential and noteworthy variation for turbidity with increasing temperature, which may result from changes in vesicle phase structure owing to changes in the strength of the surface charge of the particles with temperature [30]. Overall, these findings for particle size, zeta potential, and turbidity indicate that OA/TW40-FAV possesses increased stability over a range of temperatures, which is strongly supported by the consistently low PDI < 0.3 observed throughout the temperature study, confirming the system’s ability to maintain a homogeneous dispersion (Table S4).

#### 2.4.2. Dilution Stability

**Figure 7:**
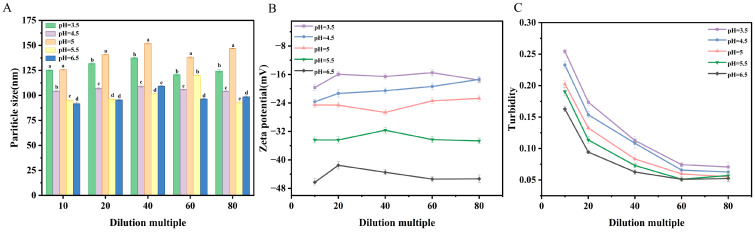
(**A**) Variations in particle size, (**B**) zeta potential and (**C**) turbidity of OA/TW40-FAV with different dilution ratios at pH = 3.5, 4.5, 5, 5.5, 6.5. Data are expressed as the mean ± standard deviation. Different superscripts indicate statistical differences (*p* < 0.05).

Dilution strongly affects the ionic strength, pH, and molecule concentration of the medium around the vesicles, which directly impacts the mechanism of assembling vesicles and size distribution, as well as encapsulation efficiency of drugs. In Figure 7A,B, the size of particles shows few changes with different dilution factors, and changes in zeta potential values were extremely minor. Critically, the system maintained a low polydispersity index (PDI < 0.3) across all dilutions, confirming that the narrow size distribution was preserved without significant aggregation or disintegration (Table S5). Figure 7C shows a steady reduction in turbidity with a rise in dilution. It may indicate that the OA/TW40 composite vesicles’ stability remains high under different dilution conditions.

#### 2.4.3. Salt Concentration Stability

**Figure 8:**
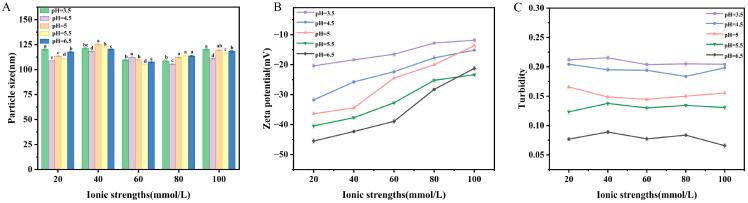
(**A**) Variations in particle size, (**B**) zeta potential and (**C**) turbidity of OA/TW40-FAV in NaCl solution with different concentrations (20–100 mM) at pH = 3.5, 4.5, 5, 5.5, 6.5. Data are expressed as the mean ± standard deviation. Different superscripts indicate statistical differences (*p* < 0.05).

Stability of vesicles at different salt concentrations was studied with respect to their ion-dependent formation mechanism and membrane charge sensitivity, which directly influence vesicle structure and size, as well as encapsulation efficiency of drugs [31]. No notable variation was noted for particle size or turbidity with rising concentration of sodium chloride (NaCl) (Figure 8A,C). The system maintained excellent dispersion stability across all tested NaCl concentrations, as evidenced by consistently low polydispersity index (PDI) values below 0.3 (Table S6). Figure 8B, however, shows a decreasing trend for the absolute value of zeta potential with an increase in NaCl concentration. This behavior can be attributed to cation-charge interaction with the negatively charged vesicular surface, which leads to compression of the electrical double layer around vesicles as well as salt-bridge equilibrium disruption. Particle aggregation, however, was not noted. These findings suggest that OA/TW40-FAVs are more stable at a variety of salt concentrations.

#### 2.4.4. Storage Time Stability

**Figure 9:**
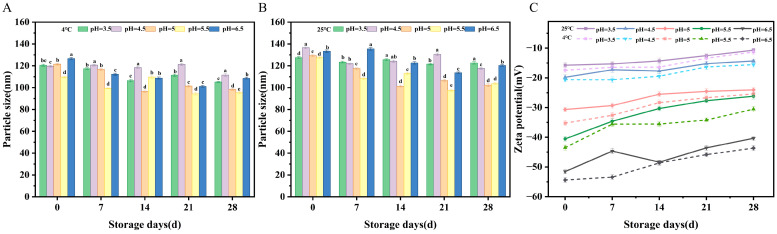
Changes in particle size and zeta potential of OA/TW40/LUT composite vesicles during 28-day storage at different pH values. (**A**) At 4 °C; (**B**) at 25 °C; (**C**) corresponding zeta potential changes. Data are expressed as the mean ± standard deviation. Different superscripts indicate statistical differences (*p* < 0.05).

Stability of vesicles with storage time was assessed, which expresses the significant need for maintaining structural integrity and functional effectiveness for long time periods during drug delivery, life science studies, and industrial uses. Figure 9A,B indicate for more than a 28-day time span, at 25 °C, the mean vesicle particle size was bigger in comparison with that at 4 °C, but no distinct changes were seen for zeta potential. Throughout the storage period, the system maintained excellent colloidal stability, with polydispersity index (PDI) values consistently below 0.3 at both storage temperatures, confirming the preservation of a homogeneous size distribution without significant aggregation or disintegration (Tables S7 and S8). This stability results from electrostatic repulsion at vesicles, which improves stability as well as helps maintain structural integrity for a certain time interval. From these results, it was seen that OA/TW40-FAV demonstrates good storage stability.

### 2.5. FT-IR, XRD and DSC Analysis of OA/TW40/LUT-FAV

**Figure 10:**
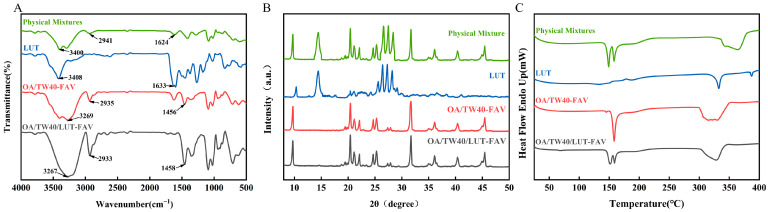
(**A**) FT-IR, (**B**) XRD and (**C**) DSC of physical mixtures, LUT, OA/TW40-FAV and OA/TW40/LUT-FAV.

FT-IR spectra of pure LUT, the physical mixture, and the OA/TW40/LUT-FAV formulation were analyzed (Figure 10A). Pure LUT showed its characteristic -C=O stretch at 1633 cm^−1^ and O-H stretch at 3408 cm^−1^. In the physical mixture, the LUT -C=O peak remained visible at 1624 cm^−1^, indicating that simple physical mixing did not alter this functional group. In the OA/TW40/LUT-FAV spectrum, the distinct LUT -C=O peak was no longer clearly detectable. Concurrently, a broad O-H band shifted from 3408 cm^−1^ to 3267 cm^−1^. These changes are consistent with the encapsulation of LUT into the vesicle matrix, where the drug likely interacts with vesicle components. The attenuation of the LUT carbonyl signal may result from its incorporation into the vesicle structure, potential dilution, and interactions such as hydrogen bonding with the OA/TW40-FAV polar groups. This interpretation is supported by the observed O-H band shift. The OA/TW40-FAV carrier exhibited an O-H stretch at 3269 cm^−1^, which shifted slightly to 3267 cm^−1^ in the OA/TW40/LUT-FAV formulation, while the -CH peaks remained stable (2933 cm^−1^ and 1458 cm^−1^). These subtle shifts further indicate molecular interactions between the carrier and LUT, such as hydrogen bonding [32]. In summary, the FT-IR data provide supporting evidence for successful LUT encapsulation and molecular interaction within the OA/TW40-FAV system. These findings align with the amorphous state of LUT indicated by XRD analysis, together confirming successful drug incorporation and a modified physical state.

In Figure 10B, LUT had various peaks of diffraction at 14.4°, 26.42°, 27.3°, and 28.2°. On the other hand, OA/TW40-FAV had a diffuse background signal lacking sharp peaks, which means that the vesicles themselves were amorphous and did not produce a crystalline diffraction pattern. Although the sharp characteristic peaks of LUT remained in the physical mixture, peak intensities were substantially reduced or completely eliminated in OA/TW40/LUT-FAV, with only broad, diffuse peaks being present. This finding indicates that LUT molecules had converted from an ordered crystalline form to an amorphous morphology upon being encapsulated in the lipophilic interior of vesicles such that they lost long-range order and failed to exhibit detectable Bragg diffraction in XRD analysis [33]. These results confirm that LUT was encapsulated within the vesicles formed by OA/TW40-FAV, rather than merely adsorbed onto or physically mixed with them.

In Figure 10C, LUT showed different melting peaks at 332 °C and 387 °C in its crystalline form. The melting peaks observed at 144 °C and 158 °C in OA/TW40-FAV correspond to characteristic peaks of the wall material. In the physical mixture sample, both the characteristic peaks of the wall material and LUT are present. However, the melting peak of LUT is absent in OA/TW40/LUT-FAV [34]. These observations suggest that LUT transitions from its crystalline state to an amorphous state upon encapsulation within the hydrophobic core of the vesicle.

### 2.6. Encapsulation and In Vitro Release Studies of OA/TW40/LUT-FAV

**Figure 11:**
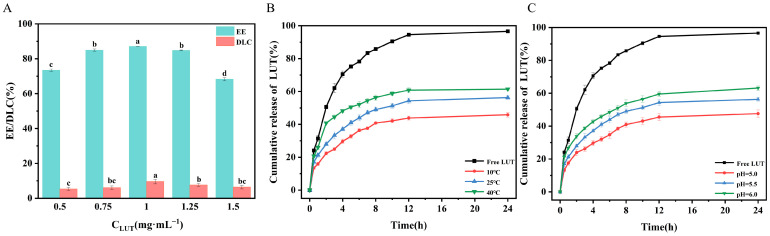
(**A**) EE and DLC of OA/TW40-FAV (10:1) as a function of luteolin concentration (0.5–1.5 mg·mL^−1^). (**B**) Release profiles of OA/TW40/LUT-FAV using LUT as a model drug under stimulation at different temperatures (pH = 5.5). (**C**) Release profiles of OA/TW40/LUT-FAV using LUT as a model drug under stimulation at different pH levels. Data are expressed as the mean ± standard deviation. Different superscripts indicate statistical differences (*p* < 0.05).

The LUT solution exhibits a maximum ultraviolet absorption peak at a wavelength of 350 nm, and in a certain range, LUT concentration exhibits a linear relationship with absorbance with the obtained regression equation y = 96.01x − 0.1071 (R^2^ = 0.9994) (Figure S2). Encapsulation efficiency of OA/TW40/LUT-FAV was calculated by measuring EE as well as DLC in a series of different concentrations of drugs (0.5 mg/mL, 0.75 mg/mL, 1 mg/mL, 1.25 mg/mL, and 1.5 mg/mL). Results for EE and DLC at different LUT concentrations are shown in Figure 11A. EE first increased with increased concentration of drugs but gradually decreased at high concentrations. Correspondingly, DLC first increased but gradually tended to decrease. At low concentrations of LUT, binding sites for drugs at the vesicular surface were unsaturated, which caused relatively low EE and DLC. With increased concentrations of LUT, to a range of 0.5 mg/mL to 1 mg/mL, increased interactions between vesicular molecule drugs as well as hydrogen bonding at the interfacial interface led to a simultaneous increase in EE as well as DLC. Above 1 mg/mL, the intrinsic loading of vesicles was surpassed, forming free, unbound LUT as well as leading to a significant reduction in EE. On the other hand, DLC was relatively steady, with vesicular saturation. Also, excess LUT will destabilize vesicular structures, enhancing release of drugs. Among the formulations tested, OA/TW40 at a 10:1 molar ratio exhibited the highest EE for LUT at 1 mg/mL, achieving an EE of approximately 87.13%, which was significantly higher than that of the 5:1 formulation (81.29%) under the same conditions (Figure S3). The TEM analysis revealed that the structural integrity of OA/TW40-FAVs remained intact following luteolin loading, with no observable morphology changes or vesicle disruption. This morphological stability aligns with the maintained physicochemical parameters and high encapsulation efficiency, collectively suggesting that luteolin molecules are effectively accommodated within the vesicular bilayers without compromising the supramolecular structure (Figure S4). The low PDI value confirms the homogeneous dispersion and excellent colloidal stability of the vesicle system (Table S9). The DLC of the 10:1 formulation was about 9.58%. At this optimal ratio and concentration, the particle size of OA/TW40/LUT composite vesicles was 152.15 ± 2.03 nm, and the absolute value of the zeta potential was −28.43 ± 0.65 mV (Figure S5). Therefore, OA/TW40/LUT-FAV at a 10:1 molar ratio with an encapsulated LUT concentration of 1 mg/mL was selected for subsequent investigations.

In Figure 11B, the cumulative release rate of LUT increases with rising temperature. This behavior could be explained by increased thermal motion of molecules and greater mobility of vesicles at high temperatures, enhancing LUT release from composite vesicles. Free LUT released rapidly at 25 °C, while LUT encapsulated in OA/TW40-FAV released slowly. At 24 h, cumulative release rates of LUT at 10 °C, 25 °C, and 40 °C were 45.83%, 56.30%, and 61.40%, respectively, which were much lower than the 96.59% release for free LUT. In contrast to free LUT, OA/TW40-FAV extended a considerably longer release time of LUT, which shows its ability to produce a sustained-release behavior. These collective findings indicate that OA/TW40-FAV can effectively improve the controlled release of active ingredients, thereby enhancing its potential reliability for topical dermal applications [35].

In Figure 11C, the cumulative release rate of LUT rose with increased pH values. This result may be explained by the ionization of OA carboxyl groups (-COO^−^), which increases the hydrophilicity of vesicles as well as enlarges intermolecular distance owing to increased electrostatic repulsion at the vesicle surface. All of these factors combine to promote rapid diffusion of LUT. It was also noted that fast release of free LUT attained 96.59% after 24 h, while cumulative release rates of composite vesicles were 47.58%, 56.30%, and 63.12% at pH 5.0, 5.5, and 6.0, respectively. Figure S6 illustrates the Ritger–Peppas model for LUT release kinetics under different pH conditions, with the correlation coefficients (R^2^) of 0.0921, 0.9643, 0.9616, and 0.9791, respectively. All of these results reaffirm that OA/TW40-FAV vesicles have a sustained-release effect on LUT [36].

Table 2 and Table S10 present the fitting equations and correlation coefficients (R^2^) for OA/TW40/LUT composite vesicles and free LUT based on four different models under varying pH and temperature conditions. Comparative analysis of the R^2^ values demonstrates that the Ritger–Peppas model yields values much closer to 1 for both free LUT and OA/TW40/LUT composite vesicles across different conditions, indicating its superior consistency with the experimental data. Furthermore, the calculated *n* values of 0.27 to 0.31 from this model unequivocally suggest a Fickian diffusion release mechanism, implying that LUT is primarily released through a diffusion-controlled process from the carrier.

### 2.7. In Vitro Transdermal and Skin Retention Analysis of OA/TW40/LUT-FAV

**Figure 12:**
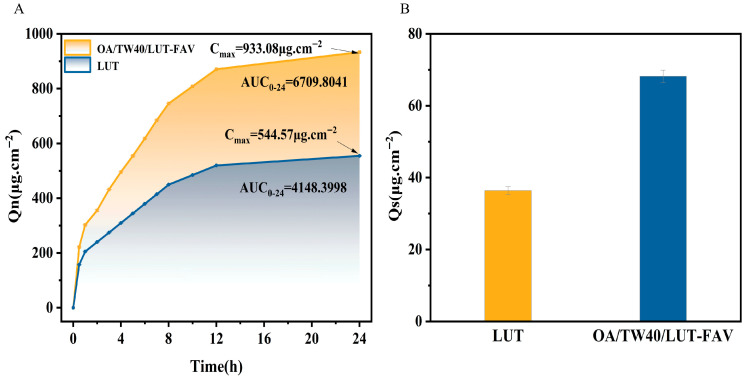
(**A**) Transdermal accumulation curve of LUT solution and OA/TW40/LUT composite vesicles. (**B**) Skin retention of LUT solution and OA/TW40/LUT composite vesicles. Data are expressed as the mean ± standard deviation.

In vitro transdermal capacity is a key parameter for evaluating topical formulations. As shown in Figure 12A and Supplementary Table S11, the cumulative drug permeation over 24 h for the OA/TW40/LUT-FAV formulation was significantly higher than that of free LUT. Specifically, the 24 h cumulative permeation of OA/TW40/LUT-FAV was 933.08 ± 1.59167 µg·cm^−2^, which was approximately double that of the free LUT solution (554.57 ± 0.75654 µg·cm^−2^). Consistent with the cumulative permeation profile, the calculated 24 h area under the curve (AUC_0–24_) for the OA/TW40/LUT-FAV group was 6709.80 µg·cm^−2^·h, significantly higher than that of the free LUT group (4148.40 µg·cm^−2^·h). Additionally, the cumulative skin retention of OA/TW40/LUT-FAV (68.19 ± 1.69060 µg·cm^−2^) was nearly double that of the free LUT control (36.39 ± 1.08666 µg·cm^−2^) (Figure 12B).

The observed enhancement in cumulative permeation and retention could be attributed to the combined physicochemical properties of the formulation. Oleic acid (OA) may promote lipid fluidity in the stratum corneum, while Tween 40 (TW40), as a surfactant, could improve drug solubility and interfacial spreading. It is plausible that their combined effect facilitates drug interaction with the skin surface [37].

Note on Interpretation: These results demonstrate the formulation’s superior performance under the experimental conditions employed. All reported permeation and retention values are cumulative amounts, expressed in consistent units of µg·cm^−2^ (or µg·cm^−2^·h for AUC), derived from the same validated dataset. However, as quantitative skin barrier integrity was not verified, the absolute flux values and the specific contribution of possible synergistic mechanisms to barrier interaction should be interpreted with this methodological consideration in mind. The findings strongly support the potential of OA/TW40/LUT-FAV for enhanced dermal delivery, but definitive mechanistic claims regarding synergy in barrier penetration require further study with validated integrity models.

### 2.8. Analysis of Antioxidant Properties of OA/TW40/LUT-FAV

#### 2.8.1. DPPH Radical Scavenging Activity

**Figure 13:**
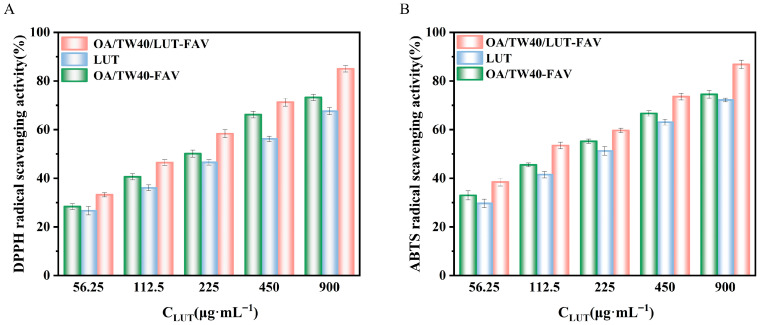
Determination of antioxidant activity of LUT solution and OA/TW40/LUT-FAV. (**A**) DPPH radical scavenging activity; (**B**) ABTS radical scavenging activity. Data are expressed as the mean ± standard deviation.

The DPPH radical scavenging activity of all tested formulations exhibited a concentration-dependent behavior (Figure 13A). Across the entire concentration range (56.25–900 µg/mL), the OA/TW40/LUT-FAV system demonstrated significantly enhanced scavenging capacity compared to both free LUT and the OA/TW40-FAV. At the highest concentration (900 µg/mL), the scavenging rates were quantified at 85.01% for OA/TW40/LUT-FAV, 73.25% for OA/TW40-FAV, and 67.57% for free LUT, respectively.

This trend was further corroborated by the IC_50_ values (Figure S7), which provided a quantitative measure of antioxidant efficacy. The OA/TW40/LUT-FAV formulation displayed the lowest IC_50_ (175.67 µg/mL), representing a substantial improvement over free LUT (471.25 µg/mL) and the blank vesicles (232 µg/mL). The superior performance of the drug-loaded vesicles is attributed to the encapsulation of LUT within the hydrophobic core of OA/TW40-FAV, which promotes stable dispersion in aqueous media and prevents molecular aggregation. This configuration enhances the accessibility of antioxidant moieties (e.g., conjugated double-bond systems) to DPPH radicals [38]. Furthermore, the vesicular structure likely shields LUT molecules from potential inactivation, thereby stabilizing their active form. Notably, the blank OA/TW40-FAV also exhibited inherent antioxidant activity, suggesting a synergistic effect between the vesicle components and the encapsulated LUT, ultimately leading to significantly potentiated antioxidant performance. These results demonstrate a synergistic effect between OA/TW40-FAV and LUT, leading to a substantial improvement in antioxidant performance.

#### 2.8.2. ABTS Free Radical Scavenging Activity

The ABTS radical scavenging activity of all tested samples showed a clear concentration-dependent enhancement (Figure 13B). Across the evaluated concentration gradient (56.25–900 µg/mL), the OA/TW40/LUT-FAV formulation consistently demonstrated superior scavenging capacity compared to both free LUT and blank OA/TW40-FAV. At the maximum tested concentration (900 µg/mL), the scavenging rates reached 86.82% for OA/TW40/LUT-FAV, 74.53% for the blank OA/TW40-FAV, and 72.18% for free LUT.

This performance hierarchy was quantitatively confirmed by IC_50_ determinations (Figure S8), which yielded values of 108.8 µg/mL for OA/TW40/LUT-FAV, 257.5 µg/mL for blank OA/TW40-FAV, and 286 µg/mL for free LUT. The significantly lower IC_50_ of the drug-loaded vesicles underscores a remarkable enhancement in antioxidant potency. The improved efficacy is attributed to the encapsulation of LUT within the OA/TW40-FAV carrier system, which promotes aqueous dispersion of the hydrophobic flavonoid in a monomolecular state. This optimal dispersion increases the effective contact area with ABTS radicals and improves the accessibility of reactive functional groups, such as phenolic hydroxyl moieties [39]. Furthermore, the vesicular architecture enables LUT to maintain high radical scavenging activity even at low concentrations (evidenced by approximately 45% scavenging at 56.25 µg/mL), thereby substantially reducing the required effective dose. Notably, the inherent antioxidant activity of the blank OA/TW40-FAV also contributes to the overall synergistic effect observed in the OA/TW40/LUT-FAV system. These findings demonstrate that the OA/TW40-FAV system can synergistically enhance the antioxidant efficacy of active ingredients, highlighting its potential as a functional delivery platform.

### 2.9. Analysis of Bacteriostatic Properties of OA/TW40/LUT-FAV

**Figure 14:**
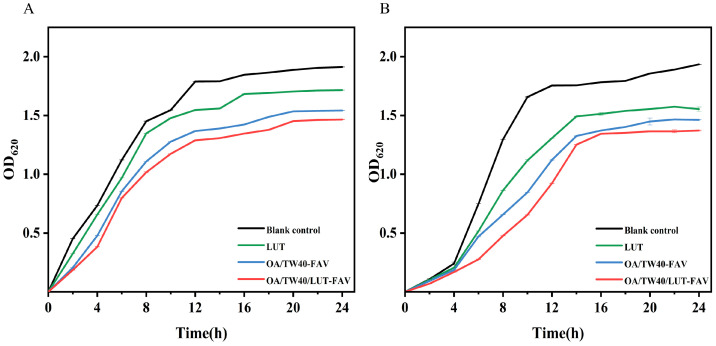
Effect of LUT, OA/TW40-FAV, and OA/TW40/LUT-FAV on the growth of *E. coli* (**A**) and *S. aureus* (**B**). Data are expressed as the mean ± standard deviation.

The growth inhibitory effects of free LUT, OA/TW40-FAV, and OA/TW40/LUT-FAV formulations against *E. coli* and *S. aureus* were monitored over 24 h via OD_620_ measurements (Figure 14A,B). Throughout the monitored period, the OA/TW40/LUT-FAV formulation consistently demonstrated stronger growth inhibition against both bacterial strains compared to the free LUT and OA/TW40-FAV controls. The observed inhibition could be attributed to several plausible mechanisms facilitated by the vesicle system. The encapsulation of LUT likely protects it from premature degradation and may enhance its local bioavailability at the bacterial interface. Furthermore, the components of the vesicle system might contribute to the overall inhibitory effect: TW40, as a surfactant, could promote interaction with bacterial membranes, while OA, an unsaturated fatty acid, might influence membrane fluidity. The combined action of these components with the released LUT suggests a potential cooperative effect against the bacteria, which may lead to more pronounced growth inhibition than with LUT alone. After 12 h, the growth curves approached a steady state, possibly indicating a sustained release profile of LUT from the vesicles [40]. In summary, under the experimental conditions tested, the OA/TW40/LUT-FAV system demonstrated stronger growth inhibition against *E. coli* and *S. aureus* compared to the controls. However, further studies quantifying minimum inhibitory concentrations (MIC) and bactericidal effects and elucidating the precise mechanism of action would be required to confirm and fully characterize its enhanced antibacterial efficacy.

## 3. Materials and Methods

### 3.1. Materials

Oleic acid (OA), luteolin (LUT) and potassium bromide (99.5% purity) were purchased from Shanghai Macklin Biochemical Co., Ltd., China. Tween 40 (TW40), DPPH and ABTS were sourced from Shanghai Aladdin Biochemical Co., Ltd., China. Anhydrous ethanol, hydrochloric acid and sodium hydroxide were purchased from Shandong Xinfar Pharmaceutical Group Chemical Reagent Co., Ltd., China. All reagents used in this experiment were analytical grade, and triple-distilled water was used throughout the investigation.

### 3.2. Preparation of OA/TW40-FAV and OA/TW40/LUT-FAV

#### 3.2.1. Preparation Procedure

A certain amount of OA (5 mmol/L) was accurately weighed and dissolved in an appropriate volume of sodium hydroxide (NaOH) solution (0.1 mol/L). The mixture was stirred at a low speed for 15 min until the solution became completely colorless and transparent, indicating the formation of sodium oleate (SO) solution. Subsequently, the SO solution was combined with TW40 and subjected to ultrasonication for 5 min at molar ratios of OA to TW40 of 5:1, 10:1, 15:1, 20:1, and 25:1, respectively. Subsequently, the mixture was stirred at a speed of 500 rpm for 1 h to guarantee uniform mixing. The pH of the mixture was subsequently adjusted with hydrochloric acid (HCl, 0.5 mol/L) under stirring, producing a set of composite vesicle solutions, which were allowed to equilibrate at room temperature for 24 h. The whole preparation was conducted at 30 °C. For preparing OA/TW40/LUT-FAV composite vesicles, LUT was weighed out and dissolved in anhydrous ethanol in concentrations of 0.5 mg/mL, 0.75 mg/mL, 1.25 mg/mL, and 1.5 mg/mL. The LUT solution was added slowly to OA/TW40 vesicles to obtain composite vesicles encapsulating LUT (OA/TW40/LUT).

#### 3.2.2. Establishment of pH Titration Curve

The pH titration was performed by gradually adding a standard HCl (or NaOH) solution to the surfactant/oleic acid mixture under vigorous stirring. This method was chosen over a simple one-point pH measurement to ensure a homogeneous and equilibrated system at each pH value, and to allow for the systematic investigation of the dynamic assembly and disassembly process of the vesicles as a function of pH. The pH was continuously monitored with a calibrated electrode throughout the titration process. A PHS-25 pH meter with an E-201C glass electrode (INESA Scientific Instrument Co., Ltd., Shanghai, China; purchased from the official authorized distributor) was employed for the accurate pH measurement of each sample. The physical appearance of each sample was visually evaluated, considering features such as colorlessness and clearness, blue opalescence, milky emulsion, and phase separation between oil and water. Afterwards, titration pH curves were plotted for both OA/TW40-FAV systems.

#### 3.2.3. Measurement of Conductivity

The pH window range is approximated to be on either side of the peak and valley of the conductivity curve [41]. Measurements were made using a DDS-308F conductivity meter (Nanjing Jiancheng Bioengineering Institute, Nanjing, China; purchased from the official authorized distributor) equipped with a glass electrode while the sample was continuously stirred, and recording began when the values stabilized.

### 3.3. Characterization of OA/TW40-FAV

The entire process was conducted at a temperature of 25 °C, with an equilibration period set to 120 s, and each sample was subjected to three parallel measurements.

#### 3.3.1. Measurement of Dynamic Light Scattering (DLS)

Measurements were carried out using a Malvern Nano ZSE (Malvern Instruments Ltd., Malvern, UK; purchased from the official authorized distributor). Before measurement, we needed to dilute the sample solution 10 times and shake well, then filter the sample through 0.45 μm filter membrane.

#### 3.3.2. Measurement of Zeta Potential

Measurements were made using a Malvern Nano ZSE (Malvern Instruments Ltd., Malvern, UK; purchased from the official authorized distributor). Before measurement, we needed to dilute the sample solution by 10 times and shake it well to avoid signal distortion caused by multiple scattering and to reduce turbidity interference [42].

#### 3.3.3. Measurement of Turbidity

The turbidity of the sample solution was determined at a wavelength of 400 nm with an ultraviolet spectrophotometer (Shimadzu Corporation, Kyoto, Japan; purchased from the official authorized distributor) [43].

#### 3.3.4. Transmission Electron Microscopy (TEM)

The morphology of OA/TW40-FAV was observed with a transmission electron microscope (TEM, Hitachi H-750, Tokyo, Japan). The OA/TW40-FAV was allowed to drop naturally at a height of 1 cm from the copper mesh, then left to stand for 1–2 min. A1–2% solution of phosphorus tungstate was added dropwise, staining the sample for 1–2 min [44].

### 3.4. OA/TW40-FAV Stability Study

In cosmetic use, stability plays a key role in guaranteeing product efficacy by retaining structural integrity of vesicles as well as efficiency in active compound delivery. With this understanding, OA/TW40-FAV was subjected to in vitro stability studies, with three concurrent measurements pertaining to each sample.

#### 3.4.1. Effect of Temperature

The chain-melting process causes temperature to directly affect the stability and functionality of vesicle membranes by altering their molecular arrangement, phase state, and interfacial forces. OA/TW40-FAV samples were subjected to constant temperatures of 20 °C, 30 °C, 40 °C, 50 °C, and 60 °C, and, following a 30 min equilibration period, measurements of particle size, zeta potential, and turbidity were conducted [45].

#### 3.4.2. Effect of Dilution Factor

In the application of OA/TW40-FAV, it is necessary to dilute the OA/TW40-FAV solution in different ratios, which will require good dilution stability of OA/TW40-FAV. The OA/TW40-FAVs were diluted 10, 20, 40, 60, and 80 times, and measurements of particle size, zeta potential, and turbidity were conducted.

#### 3.4.3. Effect of Salt Concentration

The effect of salt concentration on vesicle stability was observed through charge neutralization and disruption of the membrane structure [46]. Salt solutions at varying concentrations (20 mmol/L, 40 mmol/L, 60 mmol/L, 80 mmol/L, and 100 mmol/L) were combined with OA/TW40-FAV in a 5:1 ratio and incubated at room temperature for 12 h.

#### 3.4.4. Effect of Storage Time

One group of samples was stored at 4 °C, while the other was kept at room temperature (25 °C). Samples were collected on days 0, 7, 14, 21, and 28 to measure their particle size and zeta potential [47].

### 3.5. Fourier Transform Infrared Spectroscopy (FT-IR)

Freeze-dried samples (LUT, OA/TW40/LUT-FAV, OA/TW40-FAV and physical mixtures) were scanned from 4000 to 500 cm^−1^ using an FT-IR spectrophotometer (Vertex 70, Bruker, Germany) [48].

### 3.6. X-Ray Diffraction (XRD)

XRD patterns of different samples (LUT, OA/TW40/LUT-FAV, OA/TW40-FAV and the physical mixture) were obtained using an X-ray diffractometer (D8 advance, Bruker, Germany) at a scanning diffraction angle of 5–80° with a scan rate of 10°/min [49,50].

### 3.7. Differential Scanning Calorimetry (DSC)

A differential scanning calorimeter (DSC 3500, NETZSCH Group, Bavaria, Germany; sourced directly from the manufacturer) was used to examine the freeze-dried solids (LUT, OA/TW40/LUT-FAV, OA/TW40-FAVand the physical mixture). In the process, the samples were placed in a crucible with a nitrogen rate of 50 mL/min, a heating rate of 10 °C/min, and a heating temperature range of 20~400 °C to determine the differential scanning calorimetric curve of the samples [51].

### 3.8. Efficiency of Encapsulation

The OA/TW40/LUT-FAV mixture was adjusted to the desired concentration in the sample solution. The prepared solution was blended uniformly and treated with sonication for 10 min to promote the release of LUT. The total concentration (C_1_) was determined at 350 nm using an ultraviolet spectrophotometer, corresponding to the solute mass W_1_. Afterwards, the mixture was spun at 10,000 rpm for 30 min, after which the supernatant was diluted with ethanol to a final volume of 10 mL. The concentration of free LUT (C_2_) was analyzed, and its respective solute mass (W_2_) was calculated. The weight of freeze-dried OA/TW40/LUT-FAV was noted as W_3_ [52]. EE as well as DLC were calculated via Equations (1) and (2) below:

(1) $$EE(\%)=\frac{C_{1}-C_{2}}{C_{1}}\times 100\%$$

(2) $$DLC(\%)=\frac{W_{1}-W_{2}}{W_{3}}\times 100\%$$

### 3.9. In Vitro Release Studies

On the basis of optimal formulation, identified during preliminary experiments, an experimental sample solution of OA/TW40/LUT-FAV with a 1 mg/mL concentration was generated. Furthermore, a LUT solution of equivalent concentration was generated as a control for comparison of release behavior of drugs under different pH conditions as well as temperatures. Both the control as well as sample solutions (2 mL each) were transferred to dialysis membranes, which were safely sealed with no air bubble presence. Both dialysis membrane pouches were incubated in 200 mL PBS supplemented with 0.3% (*v*/*v*) TW80 with mild agitation that was maintained for the entire experiment. At predetermined time intervals, 3 mL samples were removed from the release medium, with an equal volume being replenished with fresh PBS [53]. The in vitro cumulative release rate was computed with Equation (3), as follows:

(3) $$Cumulative\;release(\%)=\frac{c_{n}\times V_{0}+\sum_{i=1}^{n-1}c_{i}\times V_{i}}{m}\times 100\%$$

in which Cₙ is the LUT concentration (µg·mL^−1^) in each time interval, V_0_ the solution volume (mL) in the beaker, Cᵢ the LUT concentration (µg·mL^−1^) in the solution at the (n − 1)th time point, Vᵢ the solution volume (mL) pipetted at regular intervals, and m the total LUT mass (mg) in the vesicles.

Drawing on the LUT release mechanism in Table 3 and drug release experimental data, the release kinetics of drug-loaded vesicles and LUT at 25 °C were analyzed using four models (zero-order, first-order, Higuchi, Ritger–Peppas) for mechanism interpretation and simulation; the model with R^2^ closest to 1 (i.e., maximum R^2^) was identified as optimal via regression coefficient comparison [54].

### 3.10. Percutaneous Permeability Assessment

#### 3.10.1. Skin Permeability

The skin donors used in this experiment were male Kunming mice (18–22 g), purchased from Liaoning Changsheng Biotechnology Co., Ltd., China; obtained through formal channels with complete quality inspection certificates. The experimental animal production license number SYXK (Liao) 2020-0001 and animal qualification certificate number 210726241100691448. The animal feeding and experimental procedures were in compliance with the Animal Ethics Standards of Jiamusi University, with the ethical approval number JDYXY-2024013. After euthanizing 6–8 week-old SPF-grade mice of this strain, the dorsal hair was clipped without damaging the epidermis, followed by excision of a 2 × 3 cm^2^ dorsal skin segment. Subcutaneous fat and connective tissue attached to the dermis were gently stripped using sterile ophthalmic scissors, and the isolated skin was rinsed 3 times with pre-cooled 0.9% normal saline to remove residual blood and tissue fluid. The treated mouse skin was stored in saline at 4 °C until further use. Fine skin fragments, with a diffuse surface area of 1.766 cm^2^, were positioned between the donor and receptor chambers. The receptor medium consisted of 15 mL of PBS containing 0.5% (*v*/*v*) TW80 at pH 7.0. The chamber was kept at 37 ± 0.5 °C to mimic human body temperature, while the stirring speed was fixed at 300 rpm. Immediately prior to loading, the donor formulations were filtered (0.45 μm) to remove aggregates. Subsequently, 2 mL of the OA/TW40/LUT-FAV formulation and 2 mL of the LUT solution were added to the donor chamber. At preset time intervals (0.5 h, 1 h, 2 h, 3 h, 4 h, 5 h, 6 h, 7 h, 8 h, 10 h, 12 h, 24 h), 3 mL samples were taken from the receptor chamber, which was promptly replenished with the same volume of fresh PBS. Absorbance measurements were conducted using an ultraviolet spectrophotometer, with each assay performed in triplicate [55]. The cumulative transmittance of the skin was expressed through Equation (4):

(4) $$Q_{n}=\frac{V_{0}\times c_{n}+\sum_{i=1}^{n-1}\left(c_{i}\times V_{i}\right)}{A}$$

Herein, C_n_ stands for LUT concentration (µg·mL^−1^) in each time interval, V_0_ denotes release solution volume (mL), C_i_ represents LUT concentration (µg·mL^−1^) in the solution at the (n − 1)th time point, V_i_ indicates solution volume (mL) at the (n − 1)th time point, and A stands for diffusion cell effective area (cm^2^).

#### 3.10.2. Skin Retention

The mouse skins obtained from the aforementioned experiments were rinsed with PBS solution. Subsequently, the skin samples were clipped and placed in a 10 mL beaker containing ethanol. They were then left at room temperature for 8 h to ensure complete extraction of any residual drug [56]. Finally, the absorbance was measured using an ultraviolet spectrophotometer, with each measurement performed in triplicate. The cumulative retention of the drug in the skin was obtained using Equation (5):

(5) $$Q_{m}=\frac{C_{m}\times V}{A}$$

where C_m_ is the leached drug concentration (μg·mL^−1^), V is the volume of ethanol solution (mL), and A is the transdermal permeable area (cm^2^).

#### 3.10.3. Skin Sample Quality Control and Limitation

A multi-step protocol was implemented to ensure the use of intact skin specimens in permeation studies. Each skin sample underwent (1) pre-experiment visual screening under 3× magnification to exclude tissue with visible damage; (2) post-experiment confirmation of structural integrity. Additionally, the consistency and low permeability of the control group (free LUT solution) were monitored as an indirect indicator of barrier integrity across samples.

Limitation: It should be noted that quantitative assessment of skin barrier integrity (e.g., via transepidermal water loss or electrical impedance) was not performed in this study. Therefore, while the above measures aimed to minimize variability, the permeation data may contain inherent uncertainty related to potential inter-sample differences in barrier function.

Analgesia with ibuprofen was administered when pain was anticipated; the condition of the mice was monitored daily, and if any of the following occurred—weight loss exceeding 20%, excessively large or ulcerated tumors, severe respiratory distress, paralysis, or a moribund state—euthanasia was promptly performed using CO_2_.

### 3.11. Determination of Antioxidant Activity of OA/TW40/LUT-FAV

#### 3.11.1. DPPH Free Radical Scavenging Activity

OA/TW40/LUT-FAV was diluted with a solution of LUT dissolved in anhydrous ethanol to achieve concentrations of 56.25 µg/mL, 112.5 µg/mL, 225 µg/mL, 450 µg/mL, and 900 µg/mL. A 0.1 mmol/L solution of 2,2-diphenyl-1-picrylhydrazyl (DPPH) in ethanol was prepared and used immediately. The sample group consisted of 1 mL of the sample mixed with 2 mL of the DPPH ethanol solution; the sample blank group comprised 1 mL of the sample combined with 2 mL of anhydrous ethanol; and the solvent blank group included 1 mL of anhydrous ethanol mixed with 2 mL of the DPPH ethanol solution. Subsequently, 200 µL aliquots of each sample were transferred into a 96-well plate, allowing thorough mixing in the dark at 25 °C. After a 30 min incubation, absorbance was measured at 517 nm via a microplate reader [57]. The DPPH radical scavenging activity was calculated using Equation (6):

(6) $$DPPH\;scavening\;activity\;(\%)=\frac{1-(A_{2}-A_{1})}{A_{0}}\times 100\%$$

where A_2_ is the absorbance of the sample group; A_1_ is the absorbance of the sample blank group, and A_0_ is the absorbance of the solvent blank group.

#### 3.11.2. ABTS Free Radical Scavenging Activity

A solution of 2,2′-azino-bis(3-ethylbenzothiazoline-6-sulphonic acid) (ABTS) was prepared by mixing with potassium persulfate and incubating at 25 °C for 12 h in the dark. The sample group consisted of 1 mL of the test sample combined with 4 mL of the ABTS working solution, while the blank group comprised 1 mL of triple-distilled water mixed with 4 mL of the ABTS working solution. Subsequently, 20 µL of each prepared solution was blended with 180 µL of ABTS working solution, with the reaction permitted to proceed for 8 min. Absorbance was measured at 734 nm, and all measurements were performed as described in [58]. The ABTS radical scavenging rate was determined through Equation (7):

(7) $$ABTS\;scavening\;activity\;(\%)=(\frac{A_{0}-A_{1}}{A_{0}})\times 100\%$$

where A_0_ is the absorbance of the blank group, and A_1_ is the absorbance of the sample group.

### 3.12. Antimicrobial Activity

#### 3.12.1. Microbial Strains and Culture Conditions

To visualize and quantify the antimicrobial effects of LUT, OA/TW40-FAV, and OA/TW40/LUT-FAV, *Staphylococcus aureus* (*S. aureus*) and *Escherichia coli* (*E. coli*) were employed in this study. The bacterial cultures were incubated in liquid medium at 37 °C for 24 h with continuous agitation. Prior to experimentation, bacterial concentrations were standardized to 1 × 10^6^ CFU/mL using sterile saline and the turbidity method. Additionally, all experimental glassware was sterilized at 121 °C for 15 min.

#### 3.12.2. Growth Curve

A 200 μL bacterial suspension was inoculated into 100 mL of sterile nutrient broth contained in conical flasks. To these, LUT, OA/TW40-FAV, and OA/TW40/LUT-FAV were added at a concentration of 0.1 mg/mL. A control group containing only the bacterial suspension was also established. All flasks were sealed with kraft paper and incubated in a shaking incubator at 37 °C and 200 rpm for 24 h. Absorbance at 620 nm was tested every 2 h to monitor bacterial growth, referring to the method reported by Chen et al. [59].

### 3.13. Statistical Analysis

All the data were subjected to one-way analysis of variance, and correlations between the results were carried out using Statistical Analysis System Software (IBM SPSS, Statistics 27). Significant differences were determined and accepted at *p* < 0.05.

## 4. Conclusions

In this work, an original nanocarrier system, OA/TW40-FAV, was created and optimized by a self-assembly method. TW40’s addition successfully broadened the pH window for forming vesicles from a narrow range between 8.2 and 10.08 to a wider, biologically relevant range between 3.1 and 7.2, more compatible with human skin pH. Furthermore, in vitro percutaneous penetration studies demonstrated that the combination of OA/TW40/LUT composite vesicles exhibited superior skin permeability and retention properties. These results suggested using composite vesicles as a cosmetics carrier effectively extended the continuous transdermal time of the drug. In vitro release experiments validated that OA/TW40/LUT-FAV notably improved LUT’s stability as well as its bioavailability, with sustained-release behavior as well. Additionally, the formulation presented significant antimicrobial as well as antioxidant activities. Collectively, these findings establish OA/TW40-FAV as a promising topical delivery platform for stabilizing and delivering natural active ingredients. The system’s pH compatibility with skin, enhanced penetration capability, and functional performance provide a solid foundation for its potential application in dermatologically relevant formulations. To advance toward practical implementation, future studies will focus on critical translational aspects, including formulation compatibility assessments and skin irritation evaluations.

## Figures and Tables

**Table 1:** Impact of diverse proportions on pH window expansion of OA/TW40-FAV.

OA/TW40 Different Proportions	pH Windows	Range Width
25:1	5.94–8.05	2.11
20:1	5.22–7.43	2.21
15:1	5.32–7.70	2.38
10:1	3.10–7.20	4.10
5:1	2.51–6.82	4.31

**Table 2:** Fits of four kinds of release kinetic models for LUT at different pH levels.

pH	Mode	Equation	R^2^
5	Zero order	F = 0.0172t + 0.1932	0.6355
	First order	−ln(1 − F) = 0.025t + 0.2185	0.7046
	Higuchi	F = 0.1028t^0.5^ + 0.0764	0.9029
	Ritger–Peppas	F = 19.57t^0.31^	0.9643
5.5	Zero order	F = 0.0199t + 0.2428	0.6013
	First order	−ln(1 − F) = 0.0321t + 0.2868	0.6894
	Higuchi	F = 0.1211t^0.5^ + 0.1031	0.8839
	Ritger–Peppas	F = 24.67t^0.3^	0.9616
6	Zero order	F = 0.0211t + 0.2824	0.5963
	First order	−ln(1 − F) = 0.0373t + 0.3407	0.7184
	Higuchi	F = 0.1289t^0.5^ +0.1335	0.8803
	Ritger–Peppas	F = 29.05t^0.27^	0.9791
5.5 (LUT)	Zero order	F = 0.0347t + 0.4285	0.5553
	First order	−ln(1 − F) = 0.1476t + 0.5142	0.8714
	Higuchi	F = 0.2156t^0.5^ + 0.1752	0.8510
	Ritger–Peppas	F = 43.25t^0.3^	0.9321

**Table 3:** Original and rewritten formulas for drug release kinetic modeling.

Kinetic Models	Original Formula	Rewritten Formula
Zero order	F = k_0_t	F = k_0_t
First order	F = 1 − e^−k1t^	−ln(1 − F) = k_1_t
Higuchi	F = k_H_t^0.5^	F = k_H_t^0.5^
Ritger–Peppas	F = k × (t^n^)	F = k × (t^n^)

## Data Availability

Data will be made available on request.

## Supplementary Materials

The following supporting information can be downloaded at: https://www.mdpi.com/article/10.3390/molecules31010122/s1, Figure S1: The visual appearance of samples from the pH titration curve of the OA/TW40-FAV; Figure S2: Ultraviolet spectrogram and standard curve of LUT; Figure S3: EE and DLC of OA/TW40-FAV (5:1) as a function of luteolin concentration (0.5–1.5 mg·mL^−1^); Figure S4: Particle size and zeta potential of OA/TW40/LUT composite vesicles; Figure S5: Particle size and zeta potential of OA/TW40/LUT composite vesicles; Figure S6: Ritger–Peppas model fitting for LUT release kinetics at different pH values; Figure S7: DPPH radical scavenging activity and IC_50_ values of different formulations; Figure S8: ABTS radical scavenging activity and IC_50_ values of different formulations; Table S1: PDI of the OA/TW40-FAV system under different pH conditions. Data are presented as mean ± SD (n = 3); Table S2: Zeta potential values of OA/TW40 composite solution systems at different pH values; Table S3: Turbidity values of OA/TW40 composite solution at different pH values (R = 10:1); Table S4: PDI of OA/TW40-FAV at different temperatures (20–60 °C) at pH = 3.5, 4.5, 5, 5.5, 6.5. Data are presented as mean ± SD (n = 3); Table S5: PDI of OA/TW40-FAV with different dilution ratios at pH = 3.5, 4.5, 5, 5.5, 6.5. Data are presented as mean ± SD (n = 3); Table S6: PDI of OA/TW40-FAV in NaCl solution with different concentrations (20–100 mM) at pH = 3.5, 4.5, 5, 5.5, 6.5. Data are presented as mean ± SD (n = 3); Table S7: PDI of OA/TW40/LUT composite vesicles during 28-day storage at 4 °C and varied pH. Data are presented as mean ± SD (n = 3); Table S8: PDI of OA/TW40/LUT composite vesicles during 28-day storage at 25 °C and varied pH. Data are presented as mean ± SD (n = 3); Table S9: PDI of OA/TW40/LUT composite vesicles. Data are presented as mean ± SD (n = 3); Table S10: Fits of four kinds of release kinetic models for LUT at different temperatures; Table S11: Steady-state transdermal flux of free LUT and OA/TW40/LUT-FAV in skin permeation study. Data are presented as mean ± SD (n = 3).
