# Supplementary material for: Development of Oleic Acid Composite Vesicles as a Topical Delivery System: An Evaluation of Stability, Skin Permeability, and Antioxidant and Antibacterial Activities
Source: Molecules. 2025 Dec 29;31(1):122. doi: 10.3390/molecules31010122 (PMC12786916; doi:10.3390/molecules31010122)

Table S1. PDI of the OA/TW40-FAV system under different pH conditions.Data are presented as mean ± SD (n=3)

pH	PDI ± SD
2	0.313 ± 0.005
3	0.368 ± 0.004
3.1	0.277 ± 0.006
4	0.291 ± 0.007
5	0.291 ± 0.005
6	0.292 ± 0.003
7	0.273 ± 0.004
7.2	0.286 ± 0.007
7.3	0.320 ± 0.006
8	0.360 ± 0.033
9	0.359 ± 0.007

Table S2. Zeta Potential Values of OA/TW40 Composite Solution Systems at Different pH Values.

pH	ZetaPotential ± SD (mV)
2	-10.80 ± 1.05357
3	-23.53 ± 1.00167
3.1	-31.93 ± 1.20554
4	-33.5 ± 0.8
5	-35.4 ± 0.7
6	-36.57 ± 0.97125
7	-37.33 ± 0.75056
7.2	-38.57 ± 1.04083
7.3	-45.60 ± 0.95394
8	-49.93 ± 1.24231
9	-54.77 ± 1.00167

Table S3. Turbidity Values of OA/TW40 Composite Solution at Different pH Values (R=10:1).

pH	ABS ± SD (nm)
2	1.8260 ± 0.04471
3	1.5327 ± 0.03350
3.1	0.7573 ± 0.05054
4	0.6443 ± 0.05279
5	0.6123 ± 0.05140
6	0.6033 ± 0.05227
7	0.5623 ± 0.06101
7.2	0.4430 ± 0.05903
7.3	0.1273 ± 0.00252
8	0.1130 ± 0.00200
9	0.0768 ± 0.00115

Table S4. PDI of OA/TW40-FAV with different temperature (20–60 °C) at pH = 3.5, 4.5, 5, 5.5, 6.5. Data are presented as mean ± SD (n=3)

Temperature (°C)	PDI(3.5)	PDI(4.5)	PDI(5)	PDI(5.5)	PDI(6.5)
20	0.312 ± 0.025	0.218 ± 0.009	0.216 ± 0.008	0.217 ± 0.008	0.215 ± 0.007
30	0.285 ± 0.018	0.223 ± 0.010	0.219 ± 0.009	0.221 ± 0.009	0.233 ± 0.010
40	0.241 ± 0.012	0.235 ± 0.012	0.225 ± 0.011	0.228 ± 0.011	0.258 ± 0.014
50	0.228 ± 0.009	0.251 ± 0.015	0.232 ± 0.010	0.235 ± 0.012	0.282 ± 0.017
60	0.235 ± 0.014	0.268 ± 0.018	0.240 ± 0.014	0.222 ± 0.010	0.315 ± 0.020

Table S5. PDI of OA/TW40-FAV with different dilution ratio at pH = 3.5, 4.5, 5, 5.5, 6.5. Data are presented as mean ± SD (n=3)

Dilution ratio	PDI(3.5)	PDI(4.5)	PDI(5)	PDI(5.5)	PDI(6.5)
10	0.215 ± 0.007	0.216 ± 0.007	0.221 ± 0.009	0.221 ± 0.009	0.245 ± 0.008
20	0.217 ± 0.008	0.218 ± 0.008	0.220 ± 0.009	0.222 ± 0.009	0.213 ± 0.013
40	0.219 ± 0.008	0.220 ± 0.009	0.219 ± 0.008	0.224 ± 0.010	0.244 ± 0.017
60	0.221 ± 0.009	0.222 ± 0.009	0.222 ± 0.009	0.226 ± 0.010	0.282 ± 0.011
80	0.223 ± 0.010	0.225 ± 0.010	0.223 ± 0.010	0.228 ± 0.011	0.305 ± 0.013

Table S6. PDI of OA/TW40-FAV in NaCl solution with different concentrations (20–100 mM) at pH = 3.5, 4.5, 5, 5.5, 6.5. Data are presented as mean ± SD (n=3)

Ionic strengths	PDI(3.5)	PDI(4.5)	PDI(5)	PDI(5.5)	PDI(6.5)
20	0.228 ± 0.008	0.202 ± 0.017	0.241 ± 0.009	0.271 ± 0.003	0.215 ± 0.006
40	0.235 ± 0.005	0.213 ± 0.006	0.210 ± 0.006	0.262 ± 0.005	0.213 ± 0.014
60	0.239 ± 0.007	0.217 ± 0.013	0.243 ± 0.008	0.214 ± 0.009	0.244 ± 0.007
80	0.242 ± 0.004	0.228 ± 0.015	0.232 ± 0.011	0.224 ± 0.010	0.292 ± 0.011
100	0.245 ± 0.006	0.229 ± 0.010	0.228 ± 0.010	0.228 ± 0.013	0.315 ± 0.003

Table S7. PDI of OA/TW40/LUT composite vesicles during 28-day storage under 4°C and varied pH. Data are presented as mean ± SD (n=3)

Storage days	PDI(3.5)	PDI(4.5)	PDI(5)	PDI(5.5)	PDI(6.5)
0	0.278 ± 0.018	0.233 ± 0.017	0.271 ± 0.007	0.268 ± 0.007	0.265 ± 0.007
7	0.245 ± 0.006	0.217 ± 0.008	0.208 ± 0.016	0.247 ± 0.005	0.223 ± 0.004
14	0.217 ± 0.008	0.229 ± 0.015	0.238 ± 0.012	0.228 ± 0.006	0.214 ± 0.017
21	0.253 ± 0.014	0.275 ± 0.012	0.265 ± 0.011	0.292 ± 0.010	0.245 ± 0.014
28	0.245 ± 0.010	0.283 ± 0.010	0.271 ± 0.008	0.218 ± 0.014	0.303 ± 0.013

Table S8. PDI of OA/TW40/LUT composite vesicles during 28-day storage under 25°C and varied pH. Data are presented as mean ± SD (n=3)

Storage days	PDI(3.5)	PDI(4.5)	PDI(5)	PDI(5.5)	PDI(6.5)
0	0.237 ± 0.005	0.217 ± 0.007	0.237 ± 0.008	0.261 ± 0.013	0.255 ± 0.013
7	0.228 ± 0.017	0.248 ± 0.005	0.241 ± 0.007	0.268 ± 0.005	0.213 ± 0.014
14	0.217 ± 0.013	0.233 ± 0.003	0.248 ± 0.009	0.217 ± 0.019	0.274 ± 0.007
21	0.284 ± 0.008	0.231 ± 0.005	0.255 ± 0.011	0.214 ± 0.010	0.232 ± 0.011
28	0.227 ± 0.003	0.249 ± 0.010	0.258 ± 0.010	0.229 ± 0.013	0.305 ± 0.007

Table S9. PDI of OA/TW40/LUT composite vesicles. Data are presented as mean ± SD (n=3)

C_{LUT} (mg/mL)	PDI ± SD
0.5	0.213 ± 0.006
0.75	0.227 ± 0.004
1.0	0.115 ± 0.007
1.25	0.180 ± 0.008
1.5	0.209 ± 0.003

Table S10. Fits of four kinds of release kinetic models for LUT at different temperature

Temperature（℃）	Mode	Equation	R^2
10	Zero order	$F = 0.0166t + 0.1912$	0.6103
	First order	$-ln(1-F) = 0.0238t + 0.2176$	0.6712
	Higuchi	$F =0.1006t^{0.5} + 0.0757$	0.8870
	Ritger-Peppas	$F = 19.32t^{0.31}$	0.9521
25	Zero order	$F = 0.0199t + 0.2428$	0.6013
	First order	$-ln(1-F) = 0.0321t + 0.2868$	0.6894
	Higuchi	$F = 0.1211t^{0.5} + 0.1031$	0.8839
	Ritger-Peppas	$F = 24.67t^{0.3}$	0.9616
40	Zero order	$F = 0.0197t + 0.3168$	0.4852
	First order	$-ln(1-F) = 0.0345t + 0.4065$	0.5835
	Higuchi	$F = 0.1269t^{0.5} + 0.1633$	0.7976
	Ritger-Peppas	$F = 32.6t^{0.24}$	0.9422
25(LUT)	Zero order	$F = 0.0347t + 0.4285$	0.5553
	First order	$-ln(1-F) = 0.1476t + 0.5142$	0.8714
	Higuchi	$F = 0.2156t^{0.5} + 0.1752$	0.8510
	Ritger-Peppas	$F = 43.25t^{0.3}$	0.9321

Table S11. Steady-state transdermal flux of free LUT and OA/TW40/LUT-FAV in skin permeation study. Data are presented as mean ± SD (n=3)

Time(h)	OA/TW40/LUT-FAV Qn($\mu g \cdot cm^{-2}$)	LUT Qs($\mu g \cdot cm^{-2}$)
0	0	0
0.5	221.85893 ± 0.92682	157.11342 ± 1.29587
1	302.32751 ± 0.63541	204.81045 ± 1.13573
2	355.40938 ± 3.75837	239.83988 ± 1.05226
3	431.95341 ± 0.65105	274.55849 ± 1.39051
4	495.7899 ± 1.07745	309.62311 ± 0.90096
5	554.68251 ± 0.79543	344.48833 ± 0.75654
6	618.27268 ± 1.02231	379.48257 ± 0.75654
7	684.74826 ± 1.53585	414.512 ± 0.75654
8	745.79905 ± 1.27993	449.48865 ± 0.75654
10	808.26908 ± 1.37776	484.57086 ± 0.75654
12	870.64528 ± 1.34571	519.68826 ± 0.75654
24	933.08012 ± 1.59167	554.57694 ± 0.75654

Figure S1. The visual appearance of samples from the pH titration curve of the OA/TW40-FAV

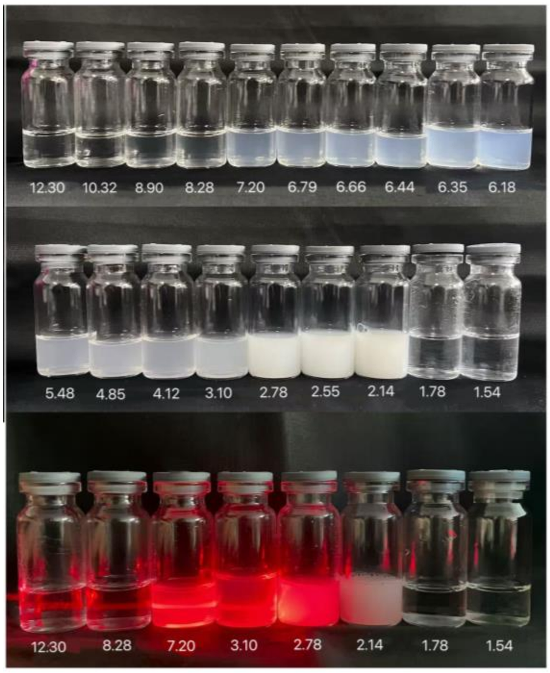

Figure S2. Ultraviolet spectrogram and Standard curve of LUT

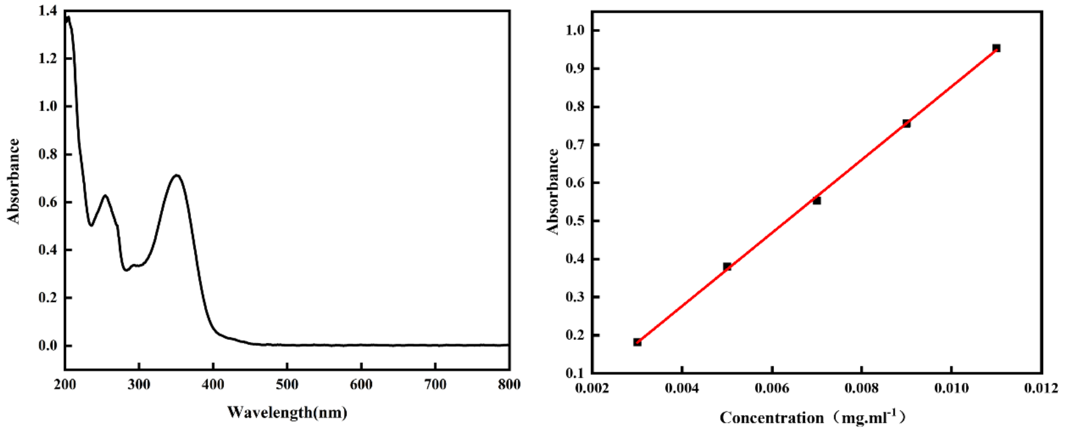

Figure S3. EE and DLC of OA/TW40-FAV (5:1) as a function of luteolin concentration (0.5–1.5 mg·mL⁻¹).

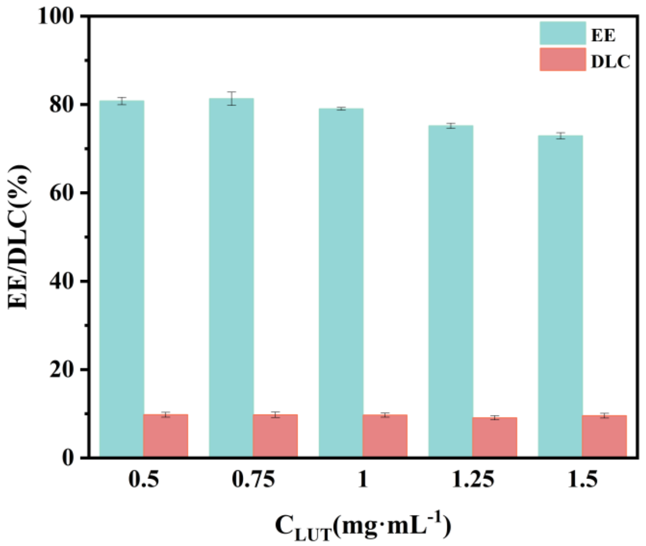

Figure S4. Particle size and Zeta potential of OA/TW40/LUT composite vesicles

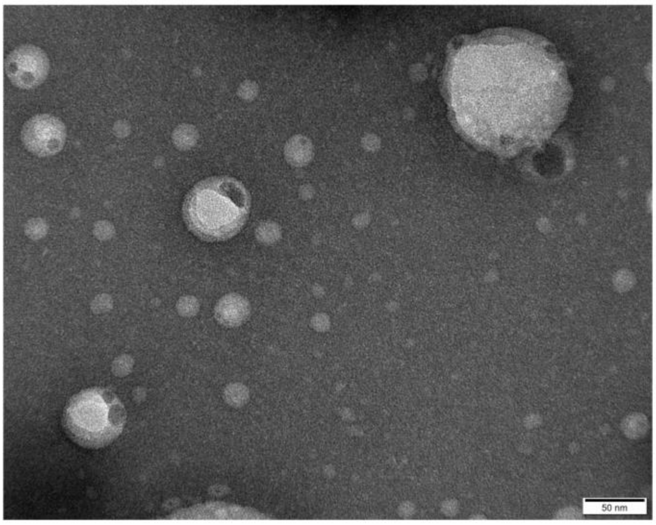

Figure S5. Particle size and Zeta potential of OA/TW40/LUT composite vesicles

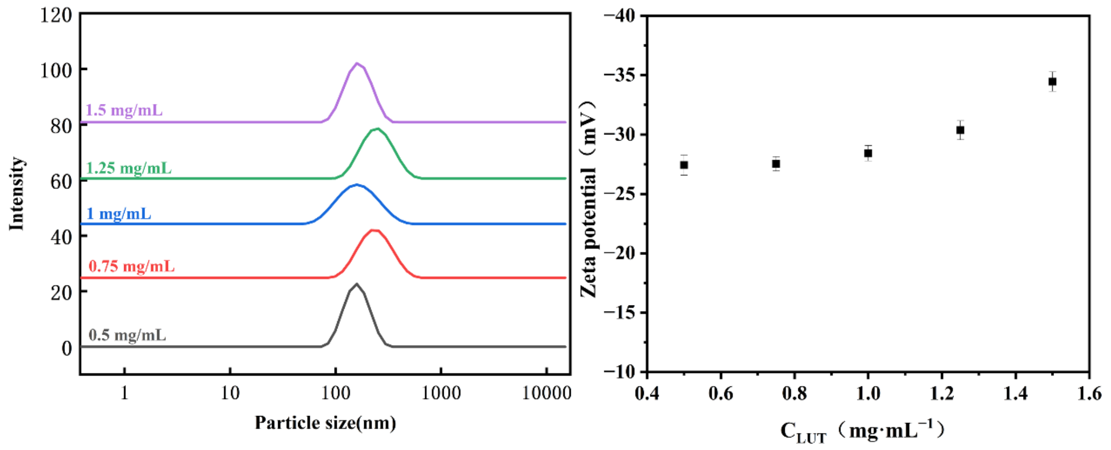

Figure S6. Ritger-Peppas model fitting for LUT release kinetics at different pH

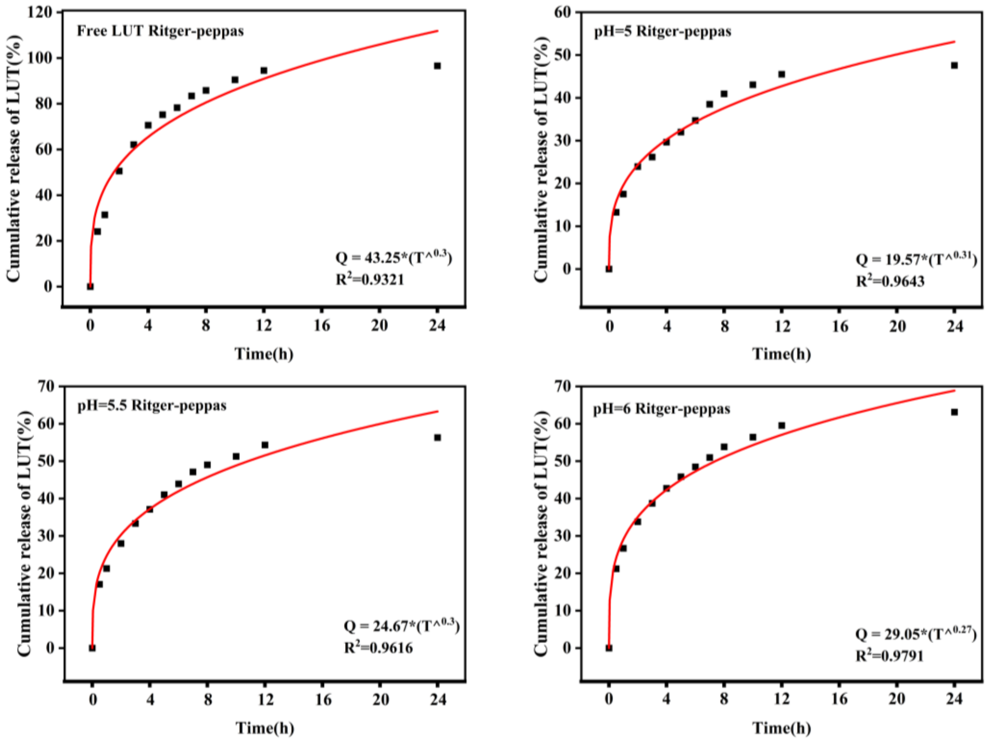

Figure S7. DPPH radical scavenging activity and IC_{50} values of different formulations

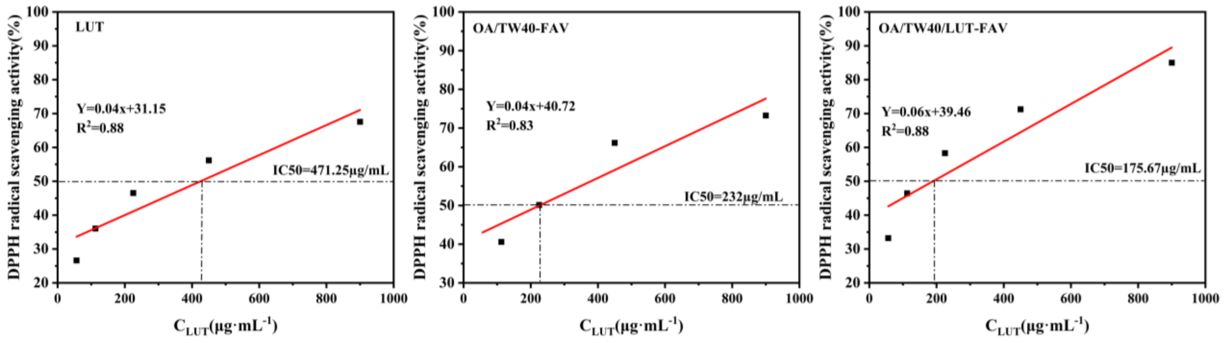

Figure S8. ABTS radical scavenging activity and IC_{50} values of different formulations

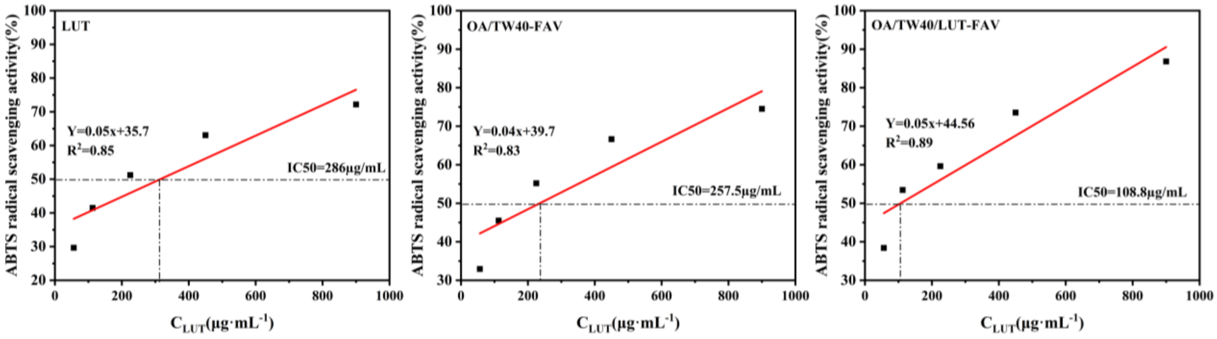

Supplement: Supplementary file 1 [file molecules-31-00122-s001.zip › molecules-3994693-supplementary.pdf]
